# Assessing junk food consumption among Australian children: trends and associated characteristics from a cross-sectional study

**DOI:** 10.1186/s12889-017-4207-x

**Published:** 2017-04-05

**Authors:** S Boylan, L. L. Hardy, B. A. Drayton, A. Grunseit, S. Mihrshahi

**Affiliations:** 1 Prevention Research Collaboration, School of Public Health, The Hub, The University of Sydney, Charles Perkins Centre D17, Level 6, Sydney, NSW 2006 Australia; 2 NSW Biostatistics Training Program, NSW Ministry of Health, Sydney, Australia

**Keywords:** Junk food, Energy-dense nutrient-poor, Children, Adolescents

## Abstract

**Background:**

The ubiquitous supply of junk foods in our food environment has been partly blamed for the increased rates in overweight and obesity. However, consumption of these foods has generally been examined individually perhaps obscuring the true extent of their combined consumption and impact on health. An overall measure of children’s junk food consumption may prove useful in the development of child obesity prevention strategies. We describe the development of a children’s Junk Food Intake Measure (JFIM) to summarise temporal change in junk food consumption and examine the association between the JFIM and health-related behaviours.

**Methods:**

Cross-sectional population surveillance survey of Australian children age 5–16 years collected in 2010 and 2015. Data were collected by questionnaire with parent’s proxy reporting for children in years K, 2 and 4 and children in years 6, 8 and 10 by self-report. Information on diet, screen-time and physical activity was collected using validated questionnaires. The JFIM comprised consumption of fried potato products, potato crisps/salty snacks, sweet and savoury biscuits/cakes/doughnuts, confectionary and, ice cream/ice blocks.

**Results:**

A total of 7565 (missing = 493, 6.1%) and 6944 (missing *n* = 611, 8.1%) children had complete data on consumption of junk foods, in 2010 and 2015, respectively. The 2015 survey data showed that among students from high socio-economic status neighbourhoods, there were fewer high junk food consumers than low junk food consumers. Children from Middle Eastern cultural backgrounds had higher junk food consumption. High junk food consumers were more likely to consume take-away ≥3/week, eat dinner in front of the television, receive sweet rewards, be allowed to consume snacks anytime, have soft drinks available at home and a TV in their bedroom. There was a lower proportion of high junk food consumers in 2015 compared to 2010.

**Conclusion:**

This is the first study to provide and examine a summary measure of overall junk food consumption among Australian children. The results indicate that junk food consumption among Australian children is lower in 2015, compared with 2010. Still, the public health workforce must continue their efforts as levels of junk food consumption remain of concern among Australian children.

## Background

In 2011, it was estimated that over one quarter (25.3%) of Australian children aged 5–17 years were overweight or obese, with 17.7% being overweight and 7.6% obese [1]. While the aetiology of these conditions may be multi-factorial, exceeding dietary energy requirements is seen as a key contributor to increasing body weight [2]. The current food environment facilitates unhealthy weight gain in children through the widespread availability, affordability and accessibility of junk foods [3]. Junk foods generally contribute few micronutrients to the diet, contain substantial amounts of fat and/or sugar and are high in energy [4]. Examples of junk foods include the majority of foods sold at fast food outlets, snack foods such as sweet and savoury biscuits and confectionery [5].

Much of the research to date examining junk food consumption has investigated junk food consumption behaviours singularly. Reporting on individual, rather than the overall or combined frequency of junk foods, may hide the true extent of junk food consumption among children and adolescents as these foods are typically eaten in combination, for example potato chips and soft drink [6]. Information on the overall rating of children’s diets may prove useful in the development of strategies aimed to reduce consumption and prevent childhood obesity.

Since the mid-1990s, attempts to assess diet quality of populations have focused on healthy eating indices or scores which provide an overall rating on a numeric scale of an individual’s dietary intake in reference to nutrient and or dietary recommendations [7]. These indices are not without their limitations, which include underutilisation among children and adolescent populations, derivation from resource intensive dietary methodology [8, 9] and little application to unhealthy eating patterns [7].

We addressed this research gap by developing a Junk Food Intake Measure (JFIM) for children and adolescents to examine the cumulative intake of junk food [10]. The aims of this study were to a) describe the development of a children’s JFIM; b) summarise junk food consumption (c) examine the association between the JFIM and health related behaviours and d) examine change in the JFIM between 2010 and 2015 among children age 5–16 years.

## Methods

### Data collection

This is a secondary data analysis of the 2010 [11] and 2015 (in press) New South Wales (NSW) Schools Physical Activity and Nutrition Survey (SPANS). SPANS is a representative cross-sectional surveillance survey of weight and weight related behaviours of children age 5–16 years living in NSW, Australia’s most populous state (2015 pop 7.6 mil) and is conducted approximately every 5 years. The surveys are school-based and use comparable sampling frames that are based on a two-stage probability sample (school and student). The probability of school selection was proportional to size of the school enrolment. Schools were sampled from each education sector (Government, Independent, Catholic) proportional to enrolment in that sector, and then all students from one to two randomly selected classes in each target grade were invited to participate. Detailed descriptions of the survey methodology are published elsewhere [12]. Briefly, the study protocols are comparable for each survey year and data are collected by trained field teams during February – April. Ethics approvals were granted by the University of Sydney Human Research Ethics Committee, the NSW Department of Education and Training (DET) and the NSW Catholic Education Commission.

### Measures

Parents of children in kindergarten, years 2 and 4 (i.e. younger children; ages 5.4–9.3 years) completed the questionnaire for their child. Children in years 6, 8 and 10 (i.e. older children; ages 11.3–15.4 years) self-completed the same questionnaire. Socio-demographic information included the child’s sex, language spoken most often at home, and postcode of residence. Postcode of residence was a proxy measure of socioeconomic status (SES) using the Australian Bureau of Statistics’ Socioeconomic Index for Areas (SEIFA) Index of Relative Socioeconomic Disadvantage [13]. SEIFA summarizes census-obtained socioeconomic indicators for geographic areas including income, educational attainment, unemployment and proportion of people in unskilled occupations. SEIFA scores from the national census most proximal to the survey year were used to rank students into low, middle, and high tertiles of SES background. Language spoken most often at home was used to categorise children into four main cultural groups according to the Australian Standard Classification of Languages [14]; English speaking, Asian, European and Middle Eastern.

Physical activity was measured by a validated one item question which asked, over the past 7-days how many days they/their child engage in moderate-to-vigorous physical activity for at least 60 min each day [15]. The response categories were 0–7 days, and dichotomised according to physical activity recommendations: 7 days (met physical activity recommendation) or <7 days (did not meet physical activity recommendation) [16, 17].

Information on screen time (television (TV), videos/DVDs, computer smart phone, tablets, e-games) was collected using a questionnaire from the Adolescent Sedentary Activity Questionnaire [18] and dichotomised for the analysis according to screen time recommendations; <2 h/day (meets recommendation) or ≥2 h/day (does not meet recommendation).

### Diet indicators

Indicators of diet were collected using a validated short food frequency questionnaire developed for population-based monitoring surveys [19]. Respondents reported consumption of fruit, vegetables, fatty meat products, red meat, fried potato products, salty snack foods, snack foods, confectionary, ice cream and beverages including sugar sweetened drinks (i.e., soft drink, diet soft drink, fruit juice), water and milk. Frequency response categories for food items were: Never or rarely, 1–2 times per week, 3–4 times per week, 5–6 times per week, 1 time per day, 2 times per day. Drinks response categories were: 1 cup or less per week, 2–4 cups per week, 5–6 cups per week, 1 cup per day, 2 cups per day (one cup defined as 250 ml).

### Junk food intake measure (JFIM)

The JFIM was based on the consumption of fried potato products (e.g. hot chips), potato crisps/salty snacks, sweet and savoury biscuits/cakes/doughnuts, confectionary and, ice cream/ice blocks. These foods are commonly consumed among Australian children [4, 20]. Each food item was assigned a score of 0–5 depending on frequency of food intake (0 being never/rarely and 5 being 2 or more times per day) so that the JFIM ranged from 0 to 25 (0 being no junk food consumed). To assess the general structure of the variables and the measure, we used principal component analysis as the data reduction technique as all the measures were discrete [21]. Principal component factor method of extraction with varimax rotation was used for all respondents with complete data. Items with loadings greater than 0.3 were used to interpret the factors. Spearman correlations were calculated between the measure and 1) anthropometric measures (waist circumference and body mass index) 2) other unhealthy food consumption (i.e., soft drink) 3) other healthy food consumption (i.e., fruit and vegetables) and 4) family food practices (soft drink availability in the home, frequency of fast food for family meals, rewarding good behaviour with sweet treats, offering water to drink with meals 5) other obesogenic behaviours (recreational screen time).

Principal component analysis generated a single factor scale with a similar structure for younger and older children with high internal consistency (Cronbach’s alpha = .744 and .749 respectively). The JFIM showed correlations in the expected direction with most variables for younger and older children. The measure was negatively and significantly correlated with fruit intake, vegetable intake and increasing frequency of the child being offered water with meals (younger children only). Similarly, positive correlations were found between the measure and soft drink and fast food consumption, increasing frequency of family meals at fast food restaurants, having sweets as a reward for good behaviour, higher soft drink availability in the home, and increasing time spent in small screen recreation.

In the current study, the JFIM was categorised into tertiles so that low, middle and high tertiles denoted scores in the ranges [0–5], [6–8], and [9–25], respectively. The JFIM was also treated as a continuous variable when examining the mean difference in the JFIM between 2010 and 2015.

### Statistical analyses

Data were analysed between January and March 2016 using SPSS Complex Sample Analysis (version 22 for Windows; IBM, Chicago, IL, USA) to account for the complex sampling design [22]. Analyses were replicated using SAS version 9.4. Post stratification weights were calculated to account for variations in response rates, along with cluster and stratification variables to account for the complex sampling design. Analyses were stratified by method of completion i.e. proxy report by parents for younger children (Years K, 2 and 4) and self-report for older children (Years 6, 8 and 10). Descriptive summaries of child characteristics by age group (i.e., younger and older) were produced for the 2010 and 2015 data and across the JFIM tertiles for the 2015 data. The mean change in the JFIM between 2010 and 2015 were also examined as well as the proportions of younger and older children in each of the JFIM tertiles in both survey years.

## Results

### Subject characteristics

In total, 8058 and 7555 children age 5–16 years participated in SPANS 2010 and 2015, respectively (response rate = 61.8% and 71%, respectively). A total of 7565 (missing = 493, 6.1%) and 6944 (missing *n* = 611, 8.1%) children had complete data on consumption of junk foods, in 2010 and 2015, respectively. The characteristics of the children are shown in Table 1. Younger children comprised of 45.4% and 50.2% of the 2010 and 2015 sample, respectively. The proportions of boys and girls for each survey year were similar, with each sex contributing to approximately half of the total sample. The majority of children were from English-speaking backgrounds in both survey years (younger: 2010 (86%), 2015 (88.4%); older: 2010 (87.4%), 2015 (87.6%). Compared with the 2010 sample, the 2015 sample had significantly more favourable behaviours with more children meeting recommended serves for fruit and for vegetables (older children only) and fewer children eating fast food regularly (older children only), eating dinner in front of the TV (younger children only), having soft drink available at home (older children only) and having a TV in their bedroom. Compared with the 2010 sample, a higher proportion of younger children consuming fast food regularly and a lower proportion of older children met the physical activity recommendation in the 2015 sample.

**Table 1:** Characteristics of the 2010 and 2015 samples (%)^1^

	Younger children (years K/2/4)		Older children (years 6/8/10)	
	2010 *n* = 3437	2015 *n* = 3489	2010 *n* = 4128	2015 *n* = 3455
Sex				
Boys	51.6	48.9	52.0	50.9
Girls	48.4	51.1	48.0	49.1
Socioeconomic tertile				
Low	29.9	20.3	28.3	31.4
Middle	43.4	33.7	38.7	33.4
High	26.7	46.0	33.0	35.2
Cultural background				
English-speaking	86.0	88.4	87.4	87.6
European	0.9	1.5	1.8	1.3
Middle Eastern	4.7	3.6	3.2	3.2
Asian	8.4	6.5	7.6	7.8
Locality				
Urban	88.3	78.4	80.1	73.7
Rural	11.7	21.6	19.9	26.3
Meets fruit serve recommendation				
No	29.9	*22.8* ^*4*^	25.0	*19.2* ^*2*^
Yes	70.1	77.2	75.0	80.8
Meets vegetable serve recommendation				
No	97.3	97.4	93.4	*88.9* ^*4*^
Yes	2.7	2.6	6.6	11.1
Takeaways ≥3/week				
No	99.2	*98.5* ^*3*^	96.8	*97.6* ^*2*^
Yes	0.8	1.5	3.2	2.4
Eat breakfast daily				
No	12.6	13.0	30.5	35.7
Yes	87.4	87.0	69.5	64.3
Eats dinner in front of the TV				
No	81.1	*84.7* ^*2*^	77.4	80.4
Yes	18.9	15.3	22.6	19.6
Sweets as a reward for good behaviour				
Rarely/never	40.1	40.8	53.9	52.1
Sometimes	49.8	52.7	39.3	42.0
Usually	10.1	6.6	6.8	5.9
Unrestricted snacking at home				
Yes	-	89.9	-	50.2
No	-	10.1	-	49.8
Soft drinks available in the home				
Rarely/never	-	66.5	31.9	*42.8* ^*4*^
Sometimes	-	27.1	40.4	41.3
Usually	-	6.5	27.7	15.9
Has TV in the bedroom				
No	76.0	*85.4* ^*2*^	60.1	*70.1* ^*4*^
Yes	24.0	14.6	39.9	29.9
Meets physical activity recommendation				
No	-	74.6	-	*87.1* ^*4*^
Yes	-	25.4	-	12.9
Meet screen time recommendation (weekdays)				
No	41.7	38.0	54.7	55.7
Yes	58.3	62.0	45.3	44.3
Meet screen time recommendation (weekend days)				
No	85.2	83.5	79.7	76.6
Yes	14.8	16.5	20.3	23.4

^1^Population-weighted proportions; Differences between 2010 and 2015 prevalences ^2^ *p* < 0.05; ^3^ *p* < 0.01; ^4^ *p* < 0.001

### Junk food intake measure

#### Distribution of the JFIM

Table 2 shows the mean frequency of food intake across the JFIM tertiles. The mean frequency for each food increases relative to each tertile and shows that a higher mean frequency of intakes in the high tertiles compared with the lower and middle tertiles.

**Table 2:** Mean (SD) food servings per week by JFIM tertile and age groups in 2015

	2015 JFIM tertile					
	Low		Middle		High	
Frequency of intake	Mean	SD	Mean	SD	Mean	SD
Younger children						
Fried potato products	0.30	0.02	0.72	0.02	1.12	0.04
Potato crisps/salty snacks	0.34	0.03	1.03	0.04	2.22	0.08
Sweet/savoury biscuits	1.00	0.03	1.73	0.04	2.73	0.06
Lollies/chocolate	0.48	0.02	1.12	0.02	1.95	0.05
Ice-cream/ice-blocks	0.72	0.03	1.32	0.02	2.22	0.04
Older children						
Fried potato products	0.33	0.02	0.84	0.02	1.41	0.04
Potato crisps/salty snacks	0.54	0.03	1.29	0.03	2.37	0.05
Sweet/savoury biscuits	0.80	0.03	1.59	0.03	2.75	0.05
Lollies/chocolate	0.42	0.02	1.13	0.02	2.30	0.05
Ice-cream/ice-blocks	0.46	0.02	1.08	0.03	1.96	0.06

#### Relationship between the JFIM and subject characteristics and behaviours

The association between children’s demographic characteristics, health behaviours and JFIM tertiles are summarised in Table 3. There were significant differences in the JFIM across SES tertiles among younger children (*p* = 0.02). Younger children from high SES neighbourhoods had a lower JFIM, compared with those from low SES neighbourhoods. Approximately half of children from Middle Eastern cultural backgrounds were high junk food consumers according to the JFIM (52.1% and 44% among younger and older students, respectively), compared with one quarter of children from European cultural backgrounds (25.9% and 23% among younger and older students, respectively; significant among younger children only *p* < 0.001).

**Table 3:** 2015 JFIM tertile prevalence by demographics and health related behaviours

	Younger Children					Older children				
		JFIM tertile					JFIM tertile			
		Low	Middle	High			Low	Middle	High	
Characteristic	n	%	%	%	*P*-value	n	%	%	%	*P*-value
Boys	1638	33.9	33.2	32.9	0.14	1737	39.4	31.8	28.8	0.72
Girls	1851	32.9	36.4	30.7		1718	40.8	30.4	28.9	
Socioeconomic tertile										
Low	679	25.4	35.8	38.8	0.02	979	37.8	29.3	32.9	0.07
Middle	1174	33.6	34.1	32.3		1232	41.3	30.6	28.1	
High	1636	36.7	34.9	28.3		1244	40.9	33.3	25.9	
Cultural background										
English-speaking	3054	34.4	34.8	30.8	<0.001	2996	40.3	31.7	28.0	0.07
European	53	48.8	25.3	25.9		47	47.4	29.7	23.0	
Middle Eastern	137	10.2	37.7	52.1		113	34.6	21.4	44.0	
Asian	216	29.0	37.4	33.6		265	38.4	29.5	32.1	
Locality										
Urban	2677	32.7	34.6	32.6	0.47	2613	39.6	30.9	29.5	0.57
Rural	812	35.7	35.6	28.6		842	41.3	31.8	27.0	
Meets fruit recommendations										
No	808	24.3	35.4	40.3	<0.001	634	31.6	32.8	35.6	<0.001
Yes	2659	36.1	34.7	29.2		2706	41.8	30.6	27.6	
Meets vegetable serve recommendation										
No	3362	33.4	34.4	32.3	0.01	2975	38.6	31.6	29.8	0.003
Yes	84	37.7	46.0	16.3		368	48.1	28.1	23.9	
Takeaways ≥3/week										
No	3431	33.8	34.9	31.3	<0.001	3345	40.8	31.3	27.9	<0.001
Yes	44	1.4	25.4	73.2		81	8.8	20.9	70.3	
Eat breakfast daily										
No	435	22.1	35.8	42.1	<0.001	1144	36.0	33.0	30.9	0.01
Yes	3031	34.9	34.6	30.4		2266	42.1	30.2	27.7	
Eats dinner in front of the TV										
No	2980	35.9	35.3	28.8	<0.001	2767	42.8	32.2	25.0	<0.001
Yes	490	19.1	32.5	48.4		655	28.4	27.1	44.5	
Sweets as a reward for good behaviour										
Rarely/never	1440	44.9	31.2	23.9	<0.001	1750	51.4	27.8	20.7	<0.001
Sometimes	1812	27.3	38.5	34.2		1433	30.2	35.5	34.3	
Usually	216	10.1	30.1	59.8		193	10.6	26.0	63.4	
Unrestricted snacking at home										
Yes	3097	14.6	30.6	54.8	<0.001	1759	28.8	33.0	38.2	<0.001
No	371	35.5	35.4	29.1		1616	51.3	29.0	19.7	
Soft drinks available in the home										
Rarely/never	2290	42.0	35.2	22.8	<0.001	1504	53.5	27.0	19.6	<0.001
Sometimes	937	17.0	36.4	46.6		1355	33.1	36.1	30.8	
Usually	235	14.5	24.9	60.6		527	22.3	28.7	49.0	
Has TV in the bedroom										
No	2985	35.4	35.1	29.5	<0.001	2401	42.0	31.6	26.4	<0.001
Yes	480	21.4	33.8	44.8		976	35.4	29.8	34.9	
Meets physical activity recommendation										
No	2588	32.5	34.9	32.5	0.04	2793	39.7	32.0	28.2	0.02
Yes	845	36.9	33.8	29.2		422	43.9	24.7	31.3	
Meets screen time recommendation (weekdays)										
No	1317	21.3	32.7	45.9	<0.001	1838	32.9	31.9	35.2	<0.001
Yes	2148	40.8	36.1	23.0		1591	48.9	30.3	20.8	
Meets screen time recommendation (weekend days)										
No	2896	29.1	36.4	34.5	<0.001	2548	35.8	32.9	31.3	<0.001
Yes	550	54.4	27.3	18.3		836	54.2	25.9	19.9	

Generally, meeting fruit and vegetable recommendations (compared with not meeting them) seemed to be significantly associated with a lower proportion of younger and older children reporting high junk food consumption (fruit: *p* < 0.001 for both younger and older children; vegetables *p* = 0.01 and *p* = 0.003 for younger and older children, respectively). Over 70% of both younger and older children who consumed takeaway three or more times per week had a high JFIM compared with approximately one-third of younger and older children who reported eating takeaway foods less frequently (*p* < 0.001). Similarly, eating dinner in front of the TV (compared with not) (*p* < 0.001), receiving sweets as a reward for good behaviour (usually compared with less frequently) (*p* < 0.001), being allowed to consume snacks at any time (*p* < 0.001), soft drinks being usually available in the home (*p* < 0.001), and having a TV in the child’s bedroom (*p* < 0.001) were all significantly associated with a higher JFIM among younger and older children. Not eating breakfast daily appeared to be associated with a higher JFIM for younger students only (*p* < 0.001). There were significant differences in the JFIM between those who did and did not meet screen time recommendations, with over half of the younger and older children who met screen time recommendations at weekends being in the low JFIM tertile (*p* < 0.001).

Compared with 2010, the proportion of low junk food consumers increased, and the proportion reporting high junk food consumers was lower in 2015 for both age groups **(**Table 4 **).** The proportions within the middle tertiles were similar between the two survey years for the two age groups. The mean JFIM were significantly lower in 2015 compared to 2010 for both younger (*p* < 0.001) and older (*p* < 0.001) children.

**Table 4:** JFIM tertile prevalence by age group in 2010 and 2015

	Younger children		Older children	
JFIM tertile	2010	2015	2010	2015
Low (%) JFIM Score 0–5	37.6	45.9	41.5	51.4
Middle (%) JFIM Score 6–8	34.1	32.1	32.3	26.4
High (%) JFIM Score 9–25	28.3	22.0	26.2	22.2
JFIM Score (mean 95% CI)	7.17 (7.05–7.30)	6.26 (5.98–6.55)	6.73 (6.40–7.06)	5.98 (5.76–6.19)
*p*-value (between survey years)	<0.001		<0.001	

## Discussion

Examining the intake of individual junk foods may hide the true extent of their consumption. Therefore, we developed the Junk Food Intake Measure (JFIM) for children and adolescents to measure the cumulative intake of junk food. The results of this study indicate that junk food consumption among school age children, measured in aggregate by the JFIM, is lower in 2015 compared with 2010. This promising downward trend may be partly due to state wide health promotion efforts [23] and the growing negative media coverage which sugar consumption, in particular, has been receiving in recent years [24, 25]. However, a recent national survey showed that on average, just over one-third (35%) of total daily energy was from ‘discretionary foods’ (junk foods) and the proportion of energy from discretionary foods was highest among 14–18 year olds (41%) [20]. In addition, this current study indicates that intakes of junk foods are still of concern among specific sub-populations such as children from Middle Eastern and low SES backgrounds.

Our analysis shows that junk food consumption is heterogeneous across a range of sociodemographic subgroups. Specifically, children from high SES neighbourhoods were more likely to have a low JFIM compared with those from low SES neighbourhoods. This is consistent with other research that shows children experiencing socioeconomic disadvantage have lower quality diets and higher intakes of junk foods and beverages [26, 27]. A recent study from NSW showed that, among low SES children, there were also clear differences in weight and weight-related behaviours according to cultural background [28]. While it has been shown that parents of children from Middle Eastern cultural backgrounds generally encouraged healthy behaviours, they also reported making regular exemptions [29]. The analysis presented here is consistent with this research, showing that approximately half of children of Middle Eastern cultural backgrounds were in the high JFIM tertile.

Watching TV while eating dinner has been associated with a lower quality diet and a higher body mass index [30, 31]. In the currently study, there were more children who eat dinner in front of the TV in the high JFIM tertile (approximately half of younger and older children), compared with the low JFIM tertile (19.1% and 28.4% younger and older children, respectively). Further, over half of younger and older children who met the screen time recommendations at weekends, were low junk food consumers. Screen time is the primary contributor to the total time spent in sedentary behaviours among young people (Biddle et al. 2014). It has been suggested that screen time, particularly TV, has an important role in the aetiology of obesity due to its relationship with other unhealthy behaviours such as snacking on junk foods, displacing physical activity and inadequate sleep [32]. In addition, advertising of junk food on TV has the potential to promote unhealthy dietary practices among children [33, 34]. The results from this current study also found that approximately 45% and 35% of younger and older children who have a TV in their bedroom, respectively, were high junk food consumers. This relationship may partly explain why children with a TV in their bedroom are also at greater risk of developing overweight and obesity [35].

We also found that a number of unhealthy parenting and home environment measures were associated with high scores on the JFIM. Despite evidence pointing towards negative long term health outcomes related to overeating and increased intake of unhealthy foods, parents commonly reward children’s behaviour with sweet foods [36–38]. Furthermore, if sweets are given as a reward food to children for eating their fruit or vegetables, children may learn to place less value on fruit and vegetables [39]. Our analysis found that approximately 60% of those who received sweets as a reward for their behaviour were high junk food consumers. In addition, 60% and 49% of younger and older children, respectively, who have soft drinks available at home, were also high junk food consumers. Such findings are of concern as frequent soft drink consumption replaces healthier beverages in the diet (such as water and milk) and may increase the risk of obesity, type 2 diabetes, dental caries, and bone fractures [40, 41]. Taken together, these associations may reflect the contribution of obesogenic household culture to unhealthy food consumption of children within these households [42]. Frequent consumption of fast food is also of concern as these foods are typically high in kilojoules, fat, saturated fat, sugar, and salt and regular fast food consumption is associated with higher caloric intake, and poorer diet quality, characterised by a diet higher in fat, carbohydrate, and sugar [43]. Over 70% of the children in the current study who reported consuming takeaways three or more times per week were high junk food consumers, again indicating that junk foods are typically eaten in combination.

### Strength and limitations

The strengths of our study include a random cluster sample, representative of NSW school age children, a relatively high response rate and the use of a JFIM. The JFIM is consistent with other health behaviours in this current analysis, confirming the tendency of these behaviours to cluster and mutually influence [44]. In addition, food frequency and descriptive terms currently used do not provide meaningful or consistent nutritional guidance [45]. Messages which more accurately reflect consumption patterns may be more effective in health promotion efforts.

Limitations to consider in the interpretation of the findings, include parent’s proxy reporting for children in Years K 2 and 4. Although parents of these children are the main providers of their child’s food, they may not necessarily be aware of foods consumed during school hours. In addition, older children, who self-reported intake in this current study, are particularly likely to misreport food intake [46]. The cross-sectional design prevents comment on causal direction. This information however is useful to determine further investigation of the associations through other research designs with temporal measures (e.g., longitudinal or pre-post intervention studies). Short questions do not accurately quantify amounts of foods consumed therefore estimates of the percentage of students meeting dietary recommendations must be interpreted with caution. However the questions used here rank individuals according to their intakes, and indicate differences in diet quality between response categories. They can also give an indication of changes in food consumption by examining the distribution of responses over time and to establish trends, provided the same survey questions are used [19].

## Conclusion

We describe a novel measure for examining overall junk food consumption. This measure is associated with a range of other health related behaviours and indicates both convergent and discriminant validity. This highlights the importance of examining clustering of food intake as assessing consumption of individual foods may not provide a complete picture of dietary health. The results of this study indicate that junk food consumption among NSW school children is lower than reported in 2010. The public health workforce must continue to roll out successful programs to address unhealthy diet behaviour and risk of overweight and obesity as levels of junk food consumption remains of concern among children.

## Abbreviations

BMI: Body mass index
FFQ: Food frequency questionnaire
JFIM: Junk food intake measure
NSW: New South Wales
SEIFA: Socioeconomic index for areas index of relative socioeconomic disadvantage
SES: Socioeconomic status
SPANS: School physical activity and nutrition survey
TV: Television
